# Low Band Gap Donor–Acceptor Type Polymers Containing 2,3-Bis(4-(decyloxy)phenyl)pyrido[4,3-b]pyrazine as Acceptor and Different Thiophene Derivatives as Donors

**DOI:** 10.3390/polym8100377

**Published:** 2016-10-24

**Authors:** Yan Zhang, Xuezhong Liu, Min Wang, Xiaoli Liu, Jinsheng Zhao

**Affiliations:** 1 Shandong Key Laboratory of Chemical Energy Storage and Novel Cell Technology, Liaocheng University, Liaocheng 252059, China; zy@lcu.edu.cn (Y.Z.); xiaoliliu1219@126.com (X.L.); 2 Gastrointestinal Surgery, Liaocheng People’s Hospital, Liaocheng 252000, China; liuxuezhong2011@126.com (X.L.); wangmin1724@163.com (M.W.)

**Keywords:** donor–acceptor type polymers, 2,3-bis(4-(decyloxy)phenyl)pyrido[4,3-b]pyrazine, electrochemical polymerization, electrochromism, near-infrared

## Abstract

Four donor–acceptor type conducting polymers, namely poly(2,3-bis(4-decyloxy)phenyl)-5,8-bis(4-thiophen-2-yl)pyrido[4,3-b]pyrazine) (P1), poly(2,3-bis(4-decyloxy)phenyl)-5,8-bis(4-butylthiophen-2-yl)pyrido[4,3-b]pyrazine) (P2), poly(2,3-bis(4-(decyloxy)phenyl)-5,8-bis(4-hexyloxythiophen-2-yl)pyrido[4,3-b]pyrazine) (P3) and poly(2,3-bis(4-(decyloxy)phenyl)-5,8-bis(2,3-dihydrothieno[3,4-b][1,4]dioxin-7-yl)pyrido[4,3-b]pyrazine) (P4), containing thiophene or its derivative as the donor and pyrido[4,3-b]pyrazine as the acceptor were prepared and characterized by cyclic voltammetry, scanning electron microscopy, and UV-Vis spectroscopy to detect the influence of the donor units’ strength on the electrochromic performances. The results demonstrated that all of the polymers could be reversibly reduced and oxidized by p-type doping and n-type doping, and showed near-infrared activities and different color changes in p-type doping process. Especially, P3 and P4 showed lower optical band gap than P1 and P2 due to the strong electron-donating hexyloxythiophen group of P3 and ethylenedioxythiophene group of P4. Besides, P3 and P4 displayed the saturated green color at the neutral state and the desirable transparency at the oxidized state. All the polymers displayed desirable optical contrasts, satisfactory coloration efficiency, excellent stability and short switching time, which made the polymers fascinating candidates in the electrochromic device applications.

## 1. Introduction

**Scheme 1:**
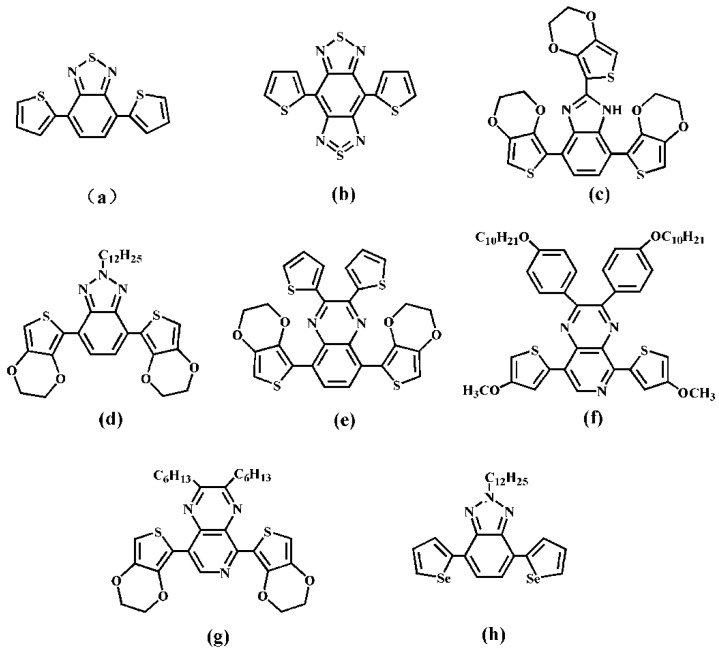
Monomer structures of some donor–acceptor (D-A) type polymers containing: (**a**) benzothiadiazole; (**b**) benzobis (thiadiazole); (**c**) benzimidazole; (**d**,**h**) benzotriazole, (**e**) quinoxaline; and (**f**,**g**) pyridopyrazine as acceptor units.

Electrochromism is defined as the reversible optical change phenomenon during the electrochemically redox process [1,2]. Recently, much attention has been paid to the electrochromic materials with near-infrared (NIR) activity such as metallopolymers [3,4], transition metal oxides WO_3_ [5] and conjugated polymers [6,7] because of the important applications in photoelectronic communication, information storage, and thermal control in spacecraft, military camouflage, etc. [8,9,10]. Among the various NIR electrochromic materials, we particularly focus on donor–acceptor (D-A) type conjugated polymers because of their low band-gap, which can lead to a spectral shift to the long wavelength into the NIR region [3,11]. D-A type polymers, which consist of alternate electron-donating and electron-withdrawing moieties, have the intra- and/or inter-molecular charge transfer (CT) bands arising from push/pull interactions between the D and A units. When the donor and acceptor match appropriately, hybridization of the electronic levels of highest occupied molecular orbital (HOMO) and lowest unoccupied molecular orbital (LUMO) leads to the ionization potential near to the donor and the electron affinity near to the acceptor which causes lower band-gap with broader absorption range, thus the polymers exhibit multiple optical properties. Scheme 1 illustrated the monomer structure of some D-A type polymers containing benzothiadiazole, benzobis(thiadiazole), benzimidazole, benzotriazole, quinoxaline or pyridopyrazine as the acceptor [12,13,14,15,16,17,18,19]. These conjugated polymers exhibit relative lower band gap with multicolor change. Designability of monomer structure is an important feature of D-A type polymers since the slight structural change of the donor or acceptor unit could lead to remarkable difference of electrochromic properties, which makes it possible to change the band gap via choosing different donor and acceptor units and achieve controllability of photoelectric properties. For instance, Reynolds group reported a series of D-A type polymers containing thiophene or its derivative as donor units for application in solar cells, organic field effect transistor (OFET) devices and polymer electrochromes, and investigated the effects of the donor unit length or the donor unit structure on the charge transport process and polymer performance [20,21,22].

Pyrido[3,4-b] pyrazine is the aromatic heterocyclic compound having the analogous molecular structure with quinoxaline (benzopyrazine), which can be used as acceptor units with the stronger electron-withdrawing ability in EC polymers due to the existence of the imine nitrogen group [23,24]. In the previous investigation, we synthesized some pyrido[3,4-b]pyrazine- or quinoxaline-based polymers containing thiophene or its derivative as as donor units [17,25,26]. A crucial factor of gaining the high-performance electrochromic polymers with low band gap was that the rigid planar structure of pyrido[3,4-b]pyrazine or quinoxaline ring can extend π electron conjugated system. On the other hand, through choosing different electron-donating units, the energy levels could be fine-tuned because of the modulated CT strength, thereby leading to conspicuous electrochromic activities in NIR region. Moreover, these polymers exhibited the n-type doping during the negative potential scan. Up to now, n-type polymers containing low band gap electron-withdrawing units such as pyrido[3,4-b]pyrazine are less studied and available than the p-type polymers though they reveal great potential in the application field of super capacitor, light emitting diode and transistor [27,28]. The major factor for this is the radical anion of the conjugated polymer produced from n-type doping process lacks stability and storage capacity at the applied reduction potential.

Based the previous research, pyrido[3,4-b]pyrazine has a stronger electron-withdrawing ability than pyridine and quinoxaline as acceptor unit in EC polymer owing to the additional pyridine N-atoms [17,23,25], herein pyrido[3,4-b]pyrazine is chosen as the acceptor unit in this contribution. As the band gap level can be changed by introducing different donors, four kinds donor units including thiophene, butylthiophen, hexyloxythiophen and 3,4-ethylenedioxythiophene are joined to pyrido[3,4-b]pyrazine groups to facilitate the charge transfer and further reduce the band gap. It can be expected that the corresponding conjugated polymers based pyrido[3,4-b]pyrazine with a gradual change in the electron-donating ability are likely to result in different optical change in visible region and NIR region with low band gap.

According to above analysis, four new D-A type monomers of 2,3-bis(4-decyloxy)phenyl)-5,8-bis(4-thiophen-2-yl)pyrido[4,3-b]pyrazine (M1), 2,3-bis(4-decyloxy)phenyl)-5,8-bis(4-butylthiophen-2-yl)pyrido[4,3-b]pyrazine (M2), 2,3-bis(4-(decyloxy)phenyl)-5,8-bis(4-hexyloxythiophen-2-yl)pyrido[4,3-b]pyrazine (M3) and 2,3-bis(4-(decyloxy)phenyl)-5,8-bis(2,3-dihydrothieno[3,4-b][1,4]dioxin-7-yl)pyrido[4,3-b]pyrazine (M4) were synthesized by Still Coupling reaction in this paper. The corresponding polymers P1, P2, P3 and P4 were synthesized by electrochemical polymerization. The substituent of 4-(decyloxy)phenyl on the pyrido[4,3-b]pyrazine aimed to enhance the solubility of the polymers. The electrochromic performances of the four polymers were studied and the results demonstrated that the different structure of electron donor units could cause various electrochemical and spectroelectrochemical property of the polymers.

## 2. Materials and Methods

### 2.1. Materials

Pyrido-3,4-diamine, 1,2-bis(4-methoxyphenyl)ethane-1,2-dione, 1-bromine decane, tetra-*n*-butylammonium bromide (TBAB), tributyl(thiophen-2-yl)stannane, tributyl(4-butylthiophen-2-yl)stannane, tributyl(4-hexyloxythiophen-2-yl)stannane, tributyl(2,3-dihydrothieno[3,4-b][1,4]dioxin-7-yl)stannane, bis(triphenylphosphine) dichloropalladium (Pd(PPh_3_)_2_Cl_2_), and tetrabutylammoniumhexafluorophosphate (TBAPF_6_) were purchased from Aldrich Chemical Co., Inc. (Milwaukee, WI, USA). Hydrobromic acid (HBr), bromine (Br_2_), sodium carbonate (Na_2_CO_3_), sodium thiosulfate (Na_2_S_2_O_3_), glacial acetic acid (HAc), potassium carbonate (K_2_CO_3_), toluene, tetrahydrofuran, dimethylformamide (DMF), dichloromethane (DCM), hexane, and acetonitrile (ACN) was purchased from Sinopharm Chemical Reagent Co., Ltd. (Shanghai, China).

### 2.2. Monomer Syntheses

#### 2.2.1. Process of Synthesis

**Scheme 2:**
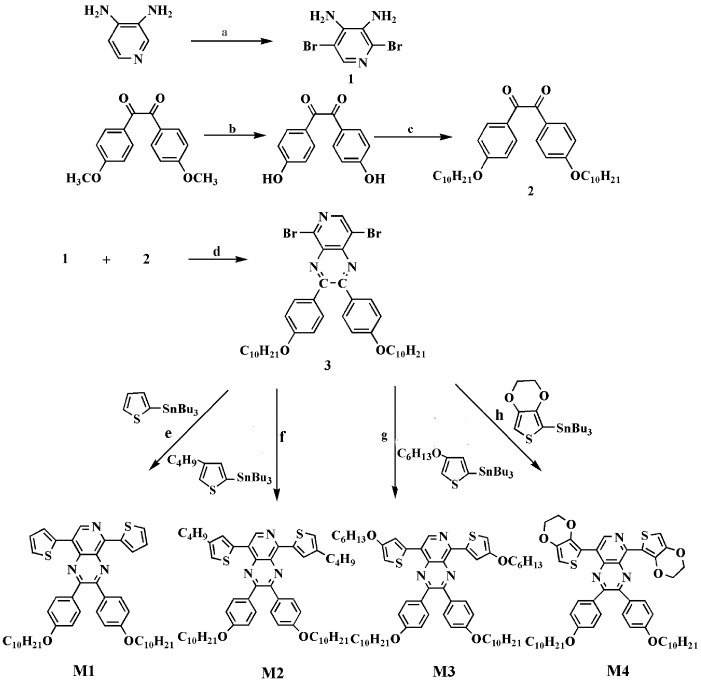
Schematic representation of syntheses of the monomers: (**a**) HBr, Br_2_, 135 °C, 5 h; (**b**) HAc, HBr, reflux, 12 h; (**c**) DMF, K_2_CO_3_, 1-bromine decane, TBAB, 120 °C, 90 min; (**d**) HAc, 50 °C, 12 h; (**e**–**h**) Pd(PPh_3_)_2_Cl_2_, tributyl(thiophen-2-yl)stannane; (**e**) tributyl(4-butylthiophen-2-yl)stannane; (**f**) tributyl(4-hexyloxythiophen-2-yl)stannane; (**g**) tributyl(2,3-dihydrothieno[3,4-b][1,4]dioxin-7-yl)stannane; (**h**) dry toluene, reflux, 24 h.

The monomer synthetic process was shown in Scheme 2. Firstly, 3,4-diamino-2,5-dibromopyridine (1) was synthetize by the bromination of 3,4-diamino pyridine at the 2 and 5 positions. Secondly, 1,2-bis(4-(decyloxy)phenyl)ethane-1,2-dione (2) was generated by the hydroxylation and alkylation of 1,2-bis(4-methoxyphenyl)ethane-1,2-dione. Thirdly, 1 reacted with 2 to obtain 5,8-dibromo-2,3-bis(4-(decyloxy)phenyl)pyrido[4,3-b]pyrazine (3). Finally, 3 reacted with corresponding tributylstannane using Pd(PPh_3_)_2_Cl_2_ as the catalytic to yield the end product M1, M2, M3 and M4, respectively.

*3,4-Diamino-2,5-dibromopyridine* (**1**). Pyrido-3,4-diamine (2 g, 18.3 mmol) and aqueous HBr (48%, 30 mL) were mixed and heated to 100 °C. After dropwise adding 2.5 mL Br_2_, the mixed solution was stirred at 135 °C for 5 h. When the reaction finished, a solution containing Na_2_CO_3_, Na_2_S_2_O_3_ and distilled water were put in the reaction solution at room temperature and a yellow deposit was gained. The product was purified according to the following order: filtration, washing by water, and recrystallization by the solution of toluene/tetrahydrofuran (*v*/*v* = 5:1). The final product 1 was white flocculence solid with the yield of 50%. ^1^H NMR (DMSO, 400 MHz, ppm): δ = 7.53 (*s*, 1H), 5.99 (*s*, 2H), 5.03 (*s*, 2H). ^13^C NMR (DMSO, 101 MHz, ppm): δ = 139.93, 139.13, 129.54, 126.67, 106.22 (see Figure S1 in Supplementary Materials).

*1,2-Bis(4-(decyloxy)phenyl)ethane-1,2-dione* (**2**). Firstly, 1,2-bis(4-methoxyphenyl)ethane-1,2-dione (3.0 g, 11 mmol), HAc (30 mL) and HBr (48%, 100 mL) were mixed orderly and heated to reflux. After stirring for 12 h and cooling to room temperature, a brown precipitation was obtained and then washed by distilled water. The product was 1,2-bis(4-hydroxyphenyl)ethane-1,2-dione with 75% yield; Secondly, 1,2-bis(4-hydroxyphenyl)ethane-1,2-dione (2.0 g, 8.2 mmol), anhydrous K_2_CO_3_ (2.4 g, 0.24 mol), 1-bromine decane (4.04 g, 3.78 mL) and tetra-*n*-butylammonium bromide (1.33 g, 4.13 mmol) were dissolved in 120 mL DMF and reacted at 120 °C for 90 min. Then after adding water and oscillating of the solution, a cream-color precipitation emerged. By filtering and washing, the product 2 was gained with about 90% productivity. ^1^H NMR (CDCl_3_, 400 MHz, ppm): δ = 7.92 (*m*, 4H, ArH), 6.94 (*m*, 4H, ArH), 4.02 (*t*, 4H), 1.80 (*m*, 4H), 1.45 (*m*, 4H), 1.33 (*m*, 24H), 0.88 (*t*, 6H). ^13^C NMR (CDCl_3_, 101 MHz, ppm): δ = 189.03, 159.95, 127.81, 121.54, 110.17, 63.94, 27.35, 25.61, 25.00, 24.75, 21.40, 18.14 (see Figure S2 in Supplementary Materials).

*5,8-Dibromo-2,3-bis(4-(decyloxy)phenyl)pyrido[4,3-b]pyrazine* (**3**). Product 1 (1 g, 3.74 mmol) and product 2 (1.955 g, 3.74 mmol) were dissolved in 60 mL HAc. The mixed solution was stirred for 12 h in argon atmosphere at 50 °C, and precipitated with yellow sediment. After filtering, washing and drying, the yellow flocculent product 3 was gained with 90% yield. ^1^H NMR (CDCl_3_, 400 MHz, ppm): δ = 8.68 (*s*, 1H), 7.67 (*m*, 4H, ArH), 6.88 (*m*, 4H, ArH), 4.00 (*t*, 4H), 1.80 (*m*, 4H), 1.42 (*m*, 28H), 0.88 (*t*, 6H). ^13^C NMR (CDCl_3_, 101 MHz, ppm): δ = 161.50, 161.18, 157.85, 156.77, 146.85, 146.00, 136.82, 132.14, 131.87, 129.80, 120.16, 114.75, 68.43, 32.11, 29.57, 26.24, 22.89, 14.32 (see Figure S3 in Supplementary Materials).

#### 2.2.2. Syntheses of M1, M2, M3 and M4

Product 3 (2 g, 2.65 mmol), tributyl(thiophen-2-yl)stannane (5.3 mmol) or tributyl(4-butylthiophen-2-yl)stannane (5.3 mmol) or tributyl(4-hexyloxythiophen-2-yl)stannane (5.3 mmol) or tributyl(2,3-dihydrothieno[3,4-b][1,4]dioxin-7-yl)stannane (5.3 mmol) and Pd(PPh_3_)_2_Cl_2_ (0.2 g, 2.85 mmol) were added in 80 mL anhydrous toluene. The mixed solution was heated up to boiling and refluxed 24 h under argon atmosphere. After evaporating the solvent of toluene under reduced pressure, the coarse products of M1, M2, M3 and M4 were obtained, respectively, which were purified by silica gel column chromatography using dichloromethane/hexane as the eluent. M1 is orange solid. ^1^H NMR (CDCl_3_, 400 MHz, ppm): δ = 9.09 (*s*, 1H), 7.90 (*s*, 2H), 7.77 (*m*, 4H, ArH), 7.54 (*s*, 2H), 7.21 (*s*, 2H), 6.92 (*m*, 4H, ArH), 3.99 (*m*, 4H), 1.71 (*m*, 4H), 1.43 (*m*, 4H), 1.37 (*m*, 24H), 0.91 (*t*, 6H). ^13^C NMR (CDCl_3_, 101 MHz, ppm): δ = 160.57, 155.49, 153.17, 151.16, 143.54, 141.45, 139.83, 136.47, 132.58, 131.96, 131.07, 130.67, 129.06, 128.02, 127.12, 126.57, 124.55, 114.60, 68.36, 32.13, 29.64, 26.22, 22.92, 14.35 (see Figure S4 in Supplementary Materials). High-resolution mass spectra (HRMS) calcd for C_47_H_57_N_3_O_2_S_2_, 759.3892; found, 759.3887. M2 is red solid. ^1^H NMR (CDCl_3_, 400 MHz, ppm): δ = 9.04 (*s*, 1H), 8.45 (*s*, 2H), 7.72 (*m*, 4H, ArH), 7.13 (*s*, 2H), 6.90 (*m*, 4H, ArH), 4.00 (*m*, 4H), 2.70 (*t*, 4H), 1.73 (*m*, 8H), 1.40 (*m*, 32H), 0.97 (*t*, 6H), 0.89 (*t*, 6H). ^13^C NMR (CDCl_3_, 101 MHz, ppm): δ = 160.55, 155.23, 152.83, 151.09, 143.97, 143.39, 140.72, 139.93, 136.14, 132.13, 131.99, 131.04, 130.71, 128.18, 126.11, 124.49, 123.95, 114.54, 68.35, 32.92, 32.13, 30.45, 29.63, 26.30, 22.91, 22.65, 14.27 (see Figure S5 in Supplementary Materials). HRMS calcd for C_55_H_73_N_3_O_2_S_2_, 871.5144; found, 871.5138. M3 is bright red solid. ^1^H NMR (CDCl_3_, 400 MHz, ppm): δ = 9.00 (*s*, 1H), 8.28 (*d*, 1H), 7.68 (*m*, 4H, ArH), 7.52 (*d*, 1H), 6.89 (*m*, 4H, ArH), 6.51 (*d*, 1H), 6.44 (*d*, 1H), 4.01 (*m*, 8H), 1.81 (*m*, 8H), 1.37 (*m*, 40H), 0.90 (*t*, 12H). ^13^C NMR (CDCl_3_, 101 MHz, ppm): δ = 160.56, 158.30, 155.45, 153.15, 153.38, 143.16, 139.96, 139.52, 135.04, 132.27, 131.87, 130.96, 130.58, 124.20, 122.75, 118.48, 114.56, 101.60, 70.48, 68.34, 32.13, 31.96, 29.63, 26.30, 25.98, 22.99, 14.32 (see Figure S6 in Supplementary Materials). HRMS calcd for C_59_H_81_N_3_O_4_S_2_, 959.5668; found, 959.5664. M4 is alsobright red solid. ^1^H NMR (CDCl_3_, 400 MHz, ppm): δ = 9.7 (*s*, 1H), 7.73 (*m*, 4H, ArH), 6.99 (*m*, 4H, ArH), 6.65 (*s*, 1H), 6.57 (*s*, 1H), 4.34 (*m*, 8H), 4.00 (*t*, 4H), 1.69 (*m*, 4H), 1.45 (*m*, 28H), 1.16 (*t*, 6H). ^13^C NMR (CDCl_3_, 101 MHz, ppm): δ = 160.76, 154.66, 152.03, 150.15, 145.00, 142.07, 141.63, 140.90, 139.29, 132.30, 132.00, 130.82, 130.61, 122.49, 114.51, 111.18, 105.81, 103.64, 68.32, 65.59, 65.07, 64.54, 32.12, 29.69, 28.01, 27.06, 26.27, 22.90, 14.34 (see Figure S7 in Supplementary Materials). HRMS calcd for C_51_H_61_N_3_O_6_S_2_, 875.4002; found, 875.3996.

### 2.3. Characterization of the Monomers and Polymers

The monomers produced in each step reaction were detected by ^1^H NMR and ^13^C NMR recorded on a Varian AMX 400 spectrometer (Varian Inc., Santa Clara, CA, USA) using tetramethylsilane (TMS) as the internal standard. High-resolution mass spectra (HRMS) were recorded on a GCT premier CAB 048 mass spectrometer (Waters Corp., Milford, MA, USA). Electrochemical syntheses and experiments were performed by a CHI760 Electrochemical workstation (Shanghai Chenhua Instrument Co., Shanghai, China) in an independent cell, employing a platinum wire (0.5 mm diameter) as working electrode (WE), a silver wire (0.03 V vs. saturated calomel electrode (SCE)) as pseudo-reference electrode (RE), and a platinum ring as counter electrode (CE). Morphology of the polymers surface was observed by a Hitachi SU-70 scanning electron microscope (SEM, Hitachi Inc., Tokyo, Japan). Spectroelectrochemical experiments was carried out in a quartz cuvette using anindium-tin-oxide-coated (ITO) glass as WE, a silver wire (0.03 V vs. SCE) as RE, and a stainless steel wire as CE, and the spectroelectrochemical properties were measured by a Varian Cary 5000 spectrophotometer (Varian Inc., Santa Clara, CA, USA). Before spectroelectrochemistry experiments, the polymer films were deposited on the Indium-tinoxide-coated (ITO) glasses (the active area: 0.9 cm × 3.0 cm). Fluorescence spectra were recorded on a Hitachi F-4600 fluorescence spectrophotometer (Hitachi Inc., Tokyo, Japan). The photographs recording different colors of the polymers were produced by a Canon Power Shot A3000 IS digital camera (Canon Inc., Tokyo, Japan).

## 3. Results and Discussion

### 3.1. Electrochemistry

**Figure 1:**
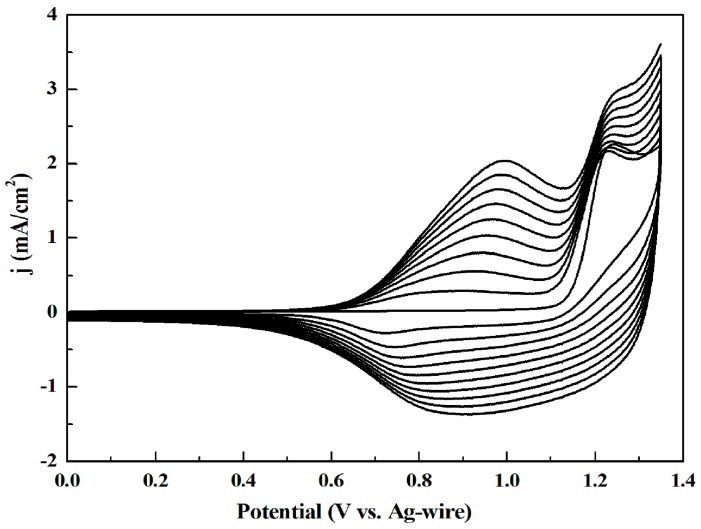
Cyclic voltammetry (CV) curves of M1.

The four polymers were produced on the working electrode through cyclic voltammetry (CV) at 100 mV/s potential scan rate in ACN/DCM mixed solution (1:1, by volume) containing 5 mM monomer and 0.1 MTBAPF_6_ as the electrolyte. Figure 1 shows the CV curves of M1, which could investigate oxidation potential and electroactivity of the monomer (the CV curves of M2, M3 and M4 are illustrated in the Supplementary Materials Figure S8a–c, respectively, with the same experimental condition). The first cycle of the voltammogram represented the oxidation of the monomer. As shown in the first cycle of the figures, the onset oxidation potentials (*E*_onset_) of M1, M2, M3 and M4 were 1.15, 1.15, 0.95 and 0.77 V, and the monomer oxidation peaks of M1, M2, M3 and M4 were 1.24, 1.26, 1.0 and 0.87 V, respectively. Compared with the other monomers, M4 had the lowest *E*_onset_ and redox potential which was caused by the strong electron-donating ability of ethylenedioxythiophene as the donor unit. The constantly increase in current density and the appearance of new redox couples with the continuing of the cyclic scan implied the deposition of conducting polymers on the electrode [29]. It can be seen from Figure 1 that M1 exhibited a reversible redox couple with broad reduction process, which correlated with the reversible p-type doping property. M2, M3 and M4 owned a similar condition with M1. The reversible p-type doping, where doping and de-doping are shown by the peaks at 0.99 and 0.88 V for M1, 1.24 and 1.08 V for M2, 0.51 and 0.76 V for M3, and 0.56 and 0.48 V for M4, respectively. M4 also showed the lowest potentials than other monomers, which was consistent with the previous results.

Electrochemical characteristics of the polymers (deposited on the platinum wires by scanning the potentials three cycles) were detected by CV test at various scan rates from 300 to 100 mV/s in DCM/ACN solution containing 0.2 M TBAPF_6_ electrolyte with the scan potentials between −1.6 and 1.35 V for P1, −1.6 and 1.4 V for P2, −1.6 and 1.2 V for P3, and −1.6 and 1.0 V for P4.

**Figure 2:**
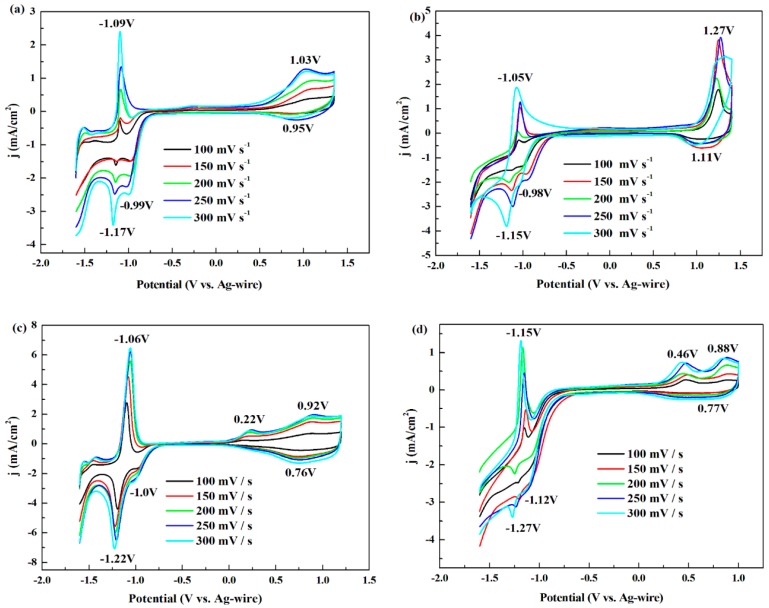
CV curves of the polymers for p-doping and n-doping processes at various scan rates: (**a**) P1; (**b**) P2; (**c**) P3; and (**d**) P4.

**Scheme 3:**
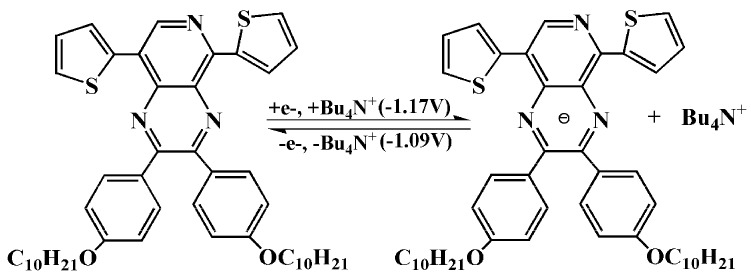
The n-type doping mechanism of P1. Bu_4_N^+^ was electrolyte cation.

As is shown in Figure 2, it can be clearly pointed out that the four polymers all had p-type doping and n-type doping process. P-type doping (or hole-transporting) polymer has been well-studied and employed as an electrode material because of the high hole mobility. However, n-type doping (or electron-transporting) process is difficult to achieve as a result of the low electron transport efficiencies and weak electronic stability [27,30]. Thus, more and more researchers devote themselves to develop high-performance n-type polymers, such as a n-type polymeric semiconductor for organic thin-film transistor (OTFT) named “ActivInk N2200” (P(NDI2OD-T2)) with electron mobility of 0.85 cm^2^/V·s produced by Polyeracorporation [31]. During the n-type doping process of P(NDI2OD-T2), the naphthalene dicarboximide (NDI) unit, as electron acceptor, was reversibly reduced by alithium addition reaction. Meanwhile, the electrons could be delocalized and stabilized by π–conjugated polymer backbone, leading to a high n-doping level [32]. In our work, P1, P2, P3 and P4 obeyed a similar n-type doping mechanism with P(NDI2OD-T2) as illustrated in Scheme 3. The reduced process occurred at pyrazine moiety of pyrido[3,4-b]pyrazine with strong electron-accepting ability owing to the two electron-withdrawing imine nitrogens in the ring. The negative charges were delocalized along the π–conjugated backbone [23,33]. It was also observed that the redox peaks of n-type doping/de-doping were much stronger than those of p-type doping/de-doping for the four polymers, which indeed proved the true occurrence of the doping process at the negative potential. Normally, the n-type doping occurs in the acceptor unit, i.e., pyridopyrazine ring, while the p-type doping occurs in the donor unit, i.e., thiophene or its derivative, which indicated that the four polymers had the strong electron acceptor and represented n-type property [34].

On the other hand, P1, P2, P3 and P4 exhibited well-defined reversible redox couple during the p-type doping and n-type doping process. According to the redox potentials and $E_{1/2}^{\mathrm{ox}}$ values of the polymers listed in Table 1, P3 and P4 exhibited relatively lower redox potentials than those of P1 and P2 in the positive and the negative scan, which was attributed to the more electron-rich hexyloxythiophen group of P3 and ethylenedioxythiophene group of P4. In addition, it was noteworthy that other potentials appeared (prepeaks) in the CV curves at −0.99 V for P1 and −0.98 V for P2 during the cathodic scan (the prepeaks in the anodic scan were insignificant). For the other two polymers, the prepeaks existed in pairs at −1.0 and 0.22 V for P3, and at −1.12 and 0.46 V for P4. This behavior was explained as charge trapping phenomenon, which means that the prepeak of n-type doping process derived from the release of the p-type doped charge that had been trapped in the polymer matrix, and the prepeak of p-type doping process came from the release of the n-type doped charge [35,36,37].

**Figure 3:**
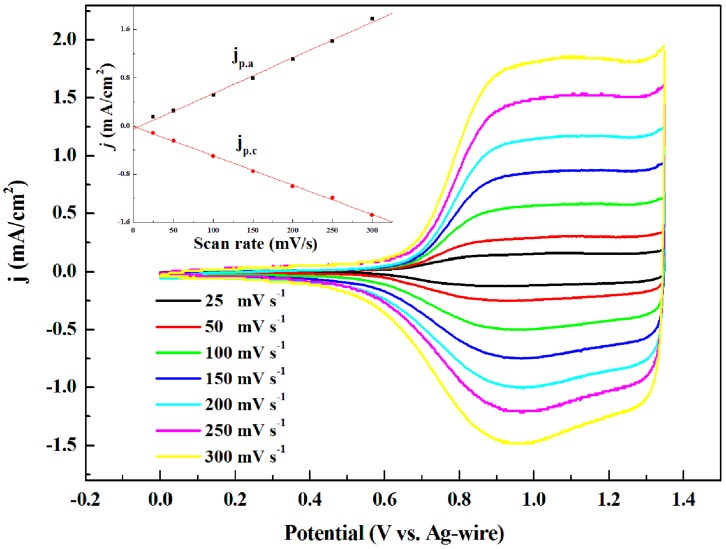
CV curves of P1 for p-type doping process at various scan rates. Insert: graphs of scan rate vs. peak current density.

The scan rate dependence of oxidation-reduction processes is aimed to research whether the process is non-diffusion controlled. The polymer films were cycled at different scan rates from 300 to 25 mV/s between the following potentials ranges: 0–1.35 V for P1 (shown in Figure 3), 0–1.4 V for P2, 0–1.2 V for P3, and −0.4–1.0 V for P4. As demonstrated in Figure 3, the current response enhanced as the scan rate increased which represented the promising electroactive and well adhered of P1 to the electrode surface. In addition, the scan rate had the good linear relation with the redox peak currents of P1, shown in the insert figure, which indicated that the electrochemical process did not displayed diffusing phenomenon even at the higher scan rates. P2, P3 and P4 had the similar curves as P1 which shown in Supplementary Materials Figure S9a–c, respectively.

**Figure 4:**
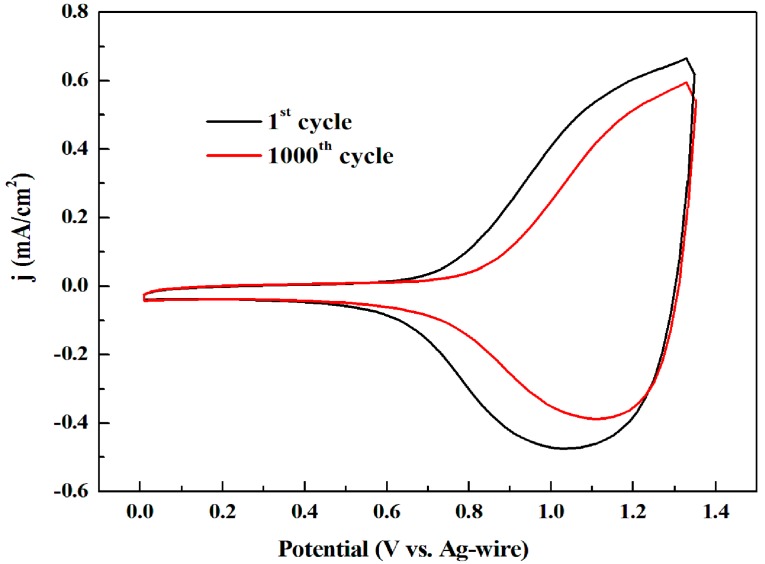
The first and the 1000th CV curve of P1.

Cyclic voltammetry provides information about the characteristics of electrochemical systems and also gives insight into kinetic properties. The long-term stability is a crucial characteristic especially on the electrochromic performance of the devices and smart windows. After the deposition on the platinum wire, the polymers were circularly scanned between neutral and oxidation states for 1000 cycles at 200 mV/s scan rate in ACN solvent with presence of 0.2 M TBAPF_6_. As shown in Figure 4, P1 retained 75% electroactivity after 1000 cycles, and P2, P3 and P4 remained higher stability with about 10% charge loss after 1000 cycles (see in Supplementary Materials Figure S10a–c, respectively). Under present experimental conditions, the four polymers displayed the satisfactory stability, which made them become potential candidates as electrochromic materials.

### 3.2. Morphology

**Figure 5:**
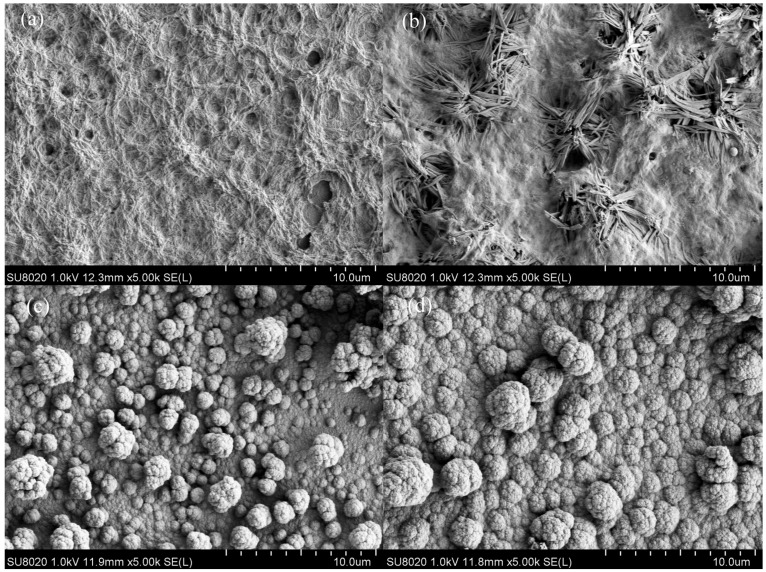
SEM images of the polymers with magnification of 5000: (**a**) P1; (**b**) P2; (**c**) P3; and (**d**) P4.

Micro-morphologies of the polymers, which might effected on their charge transport and counterions storage capability, was observed by SEM after the polymers were deposited potentiostatically on ITO glasses in the 0.2 M TBAPF_6_ACN/DCM solution with relevant monomers and de-doped. It can be found in Figure 5 that the SEM images of the four polymers were quite different with each other. The P1 film exhibited rough surface with pervasive filiform gibbosities and some holes among them (Figure 5a). The P2 film showed agglomerated coralloid cluster sembellished on the surface (Figure 5b). For P3 and P4, there were the accumulation states of clusters of globules (Figure 5c,d). Moreover, the thicknesses of the polymers, gauged by Step Profiler, were calculated as 1200, 1406, 1309 and 527 nm for P1, P2, P3 and P4, respectively (see Figure S11 in Supplementary Materials). The rough curves in the figures suggested the irregular surfaces of the films just as the above results given by the SEM images.

### 3.3. Optical Properties

**Figure 6:**
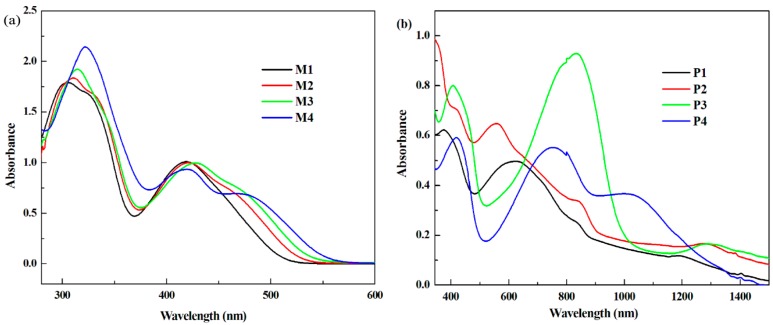
UV-vis spectra of the monomers and the polymers: (**a**) M1, M2, M3 and M4 (dissolve in DCM); and (**b**) P1, P2, P3 and P4 (deposited on ITO glasses).

The UV-vis absorption spectra of M1, M2, M3 and M4 were recorded in dichloromethane with the monomer concentration of 5 mM as shown in Figure 6a. P1, P2, P3 and P4 were deposited on ITO glasses with the charge of 9.26 C/m^2^ in DCM/ACN solution containing 5 mM monomer and 0.2 M TBAPF_6_ at 1.35, 1.4, 1.2 and 1.1 V, respectively, and then were de-doped in the same solution except without the monomer at 0, 0, 0 and −0.4 V. The UV-vis spectra of polymers on ITO glasses were investigated in solid state and showed in Figure 6b.

All the monomers showed two separate absorption bands, appearing at 306 and 419 nm for M1, 311 and 421 nm for M2, 314 and 428 nm for M3, and 322 and 420 nm for M4. The absorption peaks at about 300 nm were assigned to the strong π–π* transition in the polymer backbone, and as the lengthening of the conjugated chain from P1 to P4, the absorbed wavelength increased gradually. The absorption bands centered at more than 400 nm were attributed to the typical charge transfer band owing to thethiophene or thiophene derivative donor and pyrido[4,3-*b*]pyrazine acceptor in the conjugated structure [38,39]. The spectra of the corresponding polymers also showed two absorption bands and the peaks located at 375 and 624 nm for P1, at 415 and 556 nm for P2, at 407 and 833 nm for P3, and at 420 and 754 nm for P4. For the polymers, the absorption bands showed some bathochromic shift relative to their corresponding monomers because of the enlargement π–π* conjugation over the whole polymer backbone [25]. Another aspect was that, comparing with P1 and P2, some bathochromic shift was observed in the case of P3 and P4, which could be caused by their stronger electron donating ability and π–π* transition.

Table 2 summarizes the electrochemical parameters of the onset oxidation potential (*E*_onset_), the low energy absorption edges (λ_onset_), HOMO and LUMO energy levels, optical band gap (*E*_g,op_), etc. The calculated band gaps of the monomers (*E*_g,cal_), also listed in Table 1, were gained by the density functional theory (DFT) level using the Gaussian 09 programs. The values of *E*_g,op_ and *E*_g,cal_ of the monomers displayed the similar order followed as M1 > M2 > M3 > M4. However, for the same monomer, the value of *E*_g,cal_ was a little higher than that of *E*_g,op_, which can be explained by solvent effects or other experimental conditions. Comparing with the monomers, the corresponding polymer showed lower band gaps, especially in the case of P4 whose optical band gap was as low as 0.92 eV. This value was even lower than that of the previously reported polymer P(A) (1.14 eV) and P(B) (1.4 eV) [17,18]. This result further proved the strong electronic donating and accepting property in P4. In addition, *E*_g,ec_ was calculated from the onset potentials for p- and n-type doping of the polymers at a scan rates of 100 mV·s^−1^ in the CV curves of Figure 2. The calculated band gaps of the polymers were a certain amount higher than the optical band gaps attributed to the generation of free ions during the electrochemical experiment [40].

**Figure 7:**
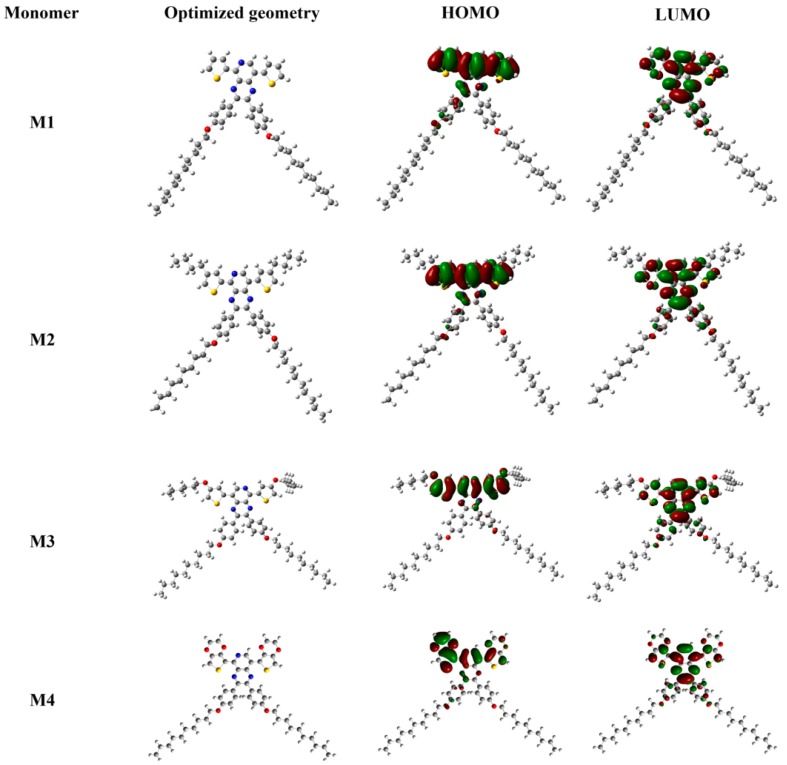
Optimized geometries and molecular orbital surfaces of the HOMOs and LUMOs for M1, M2, M3 and M4, the different colors suggested the different electronic cloud densities.

Figure 7 illustrated the electron distribution map of the HOMO and LUMO of monomers. As the figure suggests, the delocalization of the molecular orbital appeared mostly on the aromatic rings, rarely involved the external O atoms or adjacent C atoms of alkyl and alkoxy groups, and formed a large conjugated system, which guaranteed the efficient intramolecular charge transfer (ICT) process. It can also be seen from the optimized conformation that the long-chain alkoxy substituents (decyloxy) affected the planarity of the central unit to a certain degree, which would decrease the population of the pyrido[3,4-*b*] pyrazine electronic configuration. However, the alkoxy substituent could enhance the solubility of the monomers and the polymers.

**Figure 8:**
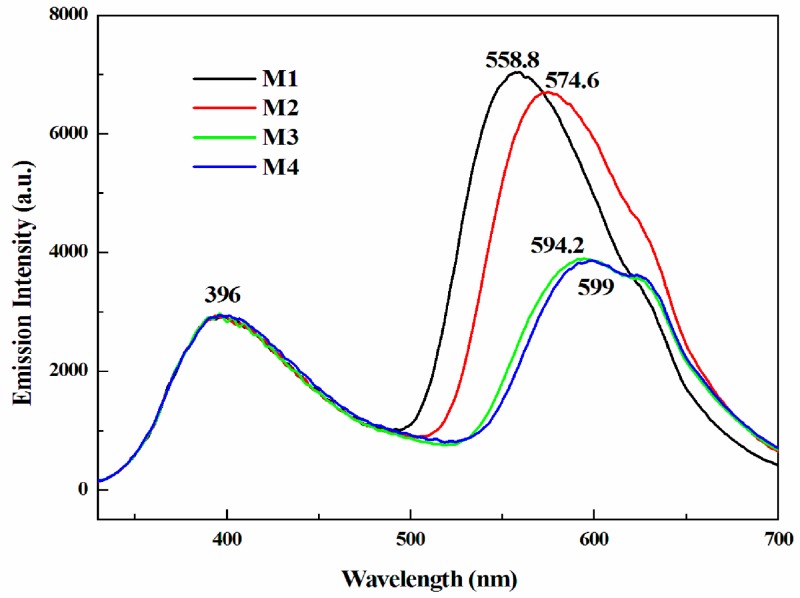
Fluorescence emission spectra of the monomers.

Fluorescence emission spectra of the monomers were recorded in DCM as shown in Figure 8. It can be observed that all the four monomers displayed distinct fluorescence character with two emission bands when excited at 315 nm. The first emission peaks of the polymers all emerged at 400 nm and the other peaks centered at different wavelength, that is, at 558.8 nm for M1, 574.6 nm for M2, 594.2 nm for M3 and 599 nm for M4. Compared with M1 and M2, the maximum emission wavelengths of M3 and M4 showed red shift, which was mainly due to the stronger electron-donating ability of hexyloxythiophen and ethylenedioxythiophene enhanced the delocalized conjugation effect.

### 3.4. Electrochromic Properties of the Polymers

The four polymers were synthesized on ITO glasses (the active area: 0.9 cm × 3.0 cm) with the same charge of 9.26 C/m^2^ and performed spectroelectrochemical test to examine the optical responses, which can prove the evolution of the charge carries in the polymer chains. The experiments of UV-vis absorption were carried out at the desired potential ranging from 0 to 1.4 V for both P1 and P2, 0 to 1.2 V for P3, and −0.4 to 1.1 V for P4 in a 0.2 M TBAPF_6_ and monomer-free ACN/DCM solution with the absorbance from 300 to 2200 nm.

**Figure 9:**
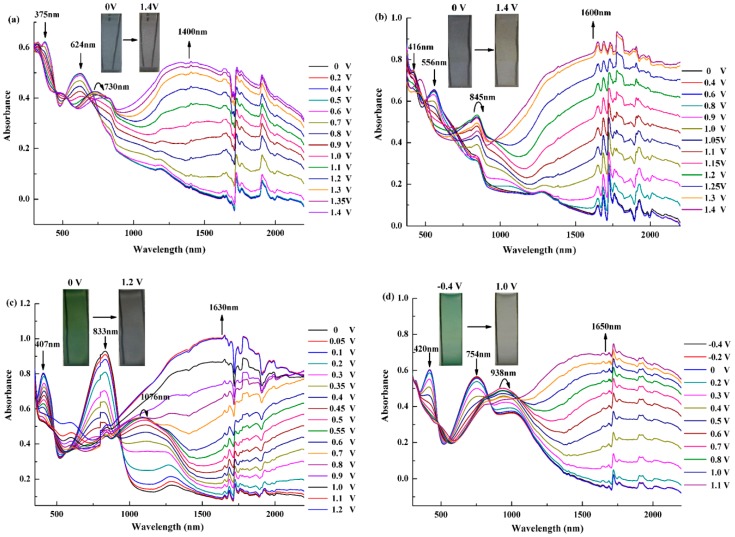
Spectroelectrochemical spectra of the polymers for p-doping process: (**a**) P1; (**b**) P2; (**c**) P3; and (**d**) P4.

Figure 9 shows the full-detailed spectroelectrochemical behavior of the polymer films and the corresponding colors. The strength of donor group not only affected the electrochemical properties but also tuned the spectroelectrochemical character of the films.

First, all four polymer films were colored at neutral state and revealed two absorption peaks. The high-energy peaks were caused by the transitions from the thiophene-based valence band to its antibonding band, while the low-energy peaks were resulted from the D-A interaction. The high-energy absorption bands of P1 and P2, centered at 375 nm for P1 and 415 nm for P2, mainly located in the UV-region, which limited the contribution to the colors. The low-energy absorption bands, centered at 624 nm for P1 and 556 nm for P2 in the visible region, made them exhibit gray blue and dark gray colors, respectively. Another important aspect was that the two coexisted absorption bands of P3 and P4 were shown in the red and blue regions, both belonging to the visible spectrum (407 and 833 nm for P3, and 420 and 754 nm for P4), which gave a green color to the polymers. In addition, π–π* interaction between donor and acceptor units could determine the match between the units because it effected the intramolecular charge transfer in the polymers. For P3 and P4, the comparable intensities of the high-energy peaks and the low-energy peaks, as shown in Figure 9c,d, suggest favorable donor–acceptor matches and strong interactions in the two polymers.

As the increasing of the applied potential for the four polymers, it can be observed that the two absorption bands at neutral state faded gradually, meanwhile, two new peaks in the NIR region intensified typically, which suggested the formation of the charge carriers attributed to the evolution of polaron and bipolaron bands. Normally, many polymers would lead tospin-1/2 polarons at light doping and blossom into spinless bipolarons at heavy doping [41]. For P1, as shown in Figure 8a, the π–π* transition bands diminished completely at 1.2 V, concurrently, a new absorption band centered at 730 nm reached the maximum owing to the formation of the polaron band. When further oxidized to 1.4 V, the other new broad band at 1400 nm attributed to bipolaron enhanced to the maximum, however, the polaronic absorption band reduced since the formation of bipolaron band caused the decrease of polaronic population, which resulted in the bleaching of the visible absorption. Similarly for others polymers, at the potential of 1.2 V for P2, 0.7 V for both P3 and P4, the π–π* transition bands disappeared while the polaron band reached a maxima, and after fully oxidation, the bipolaronic absorption peaks maximized at 1600 nm for P2, 1630 nm for P3 and 1650 nm for P4 in the NIR region. The charge carrier absorption peaks of P3 and P4 exhibited apparent bathochromic shift compared with that of P1 and P2 owing to the approving donor–acceptor matches, increasing the double-bond character and allowing absorption of low energy photons.

With the extinction of the π–π* transition bands and the emergence of the charge carrier bands, the colors were changed from gray blue to light brown for P1, from dark gray to light gray for P2, from green to transmissive light gray for P3 and P4. Especially, P3 and P4 showed the saturated green color at the neutral state and the desirable transparency at the oxidized state, which met the pivotal requirement of the ideal RGB (red, green, blue) electrochromic material. Besides, obtaining a green color was relatively difficult and rarely reported in the electrochromic literature since the polymer must simultaneously absorb in the blue and red regions under the same applied potential; fortunately, it was realized by P3 and P4 with the D-A type structure.

The three elements of color, i.e., hue, brightness and saturation, are usually measured using L*a*b* color space by CIE 1976. L* value represents lightness, a* value represents red-green balance, and b* value represents yellow-blue balance [42]. The L*, a*and b* values of the four polymers at the redox states were showed in the Table 3.

### 3.5. Electrochromic Switching

**Figure 10:**
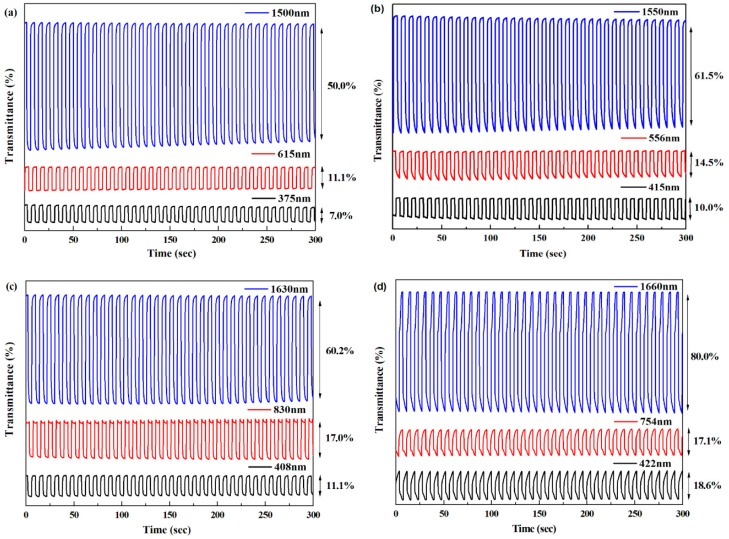
Transmittance change of the polymers with a same residence time of 4 s: (**a**) P1 monitored at 375, 615 and 1500 nm; (**b**) P2 monitored at 415, 556 and 1550 nm; (**c**) P3 monitored at 408, 830 and 1630 nm; and (**d**) P4 monitored at 422, 754 and 1660 nm.

Electrochromic switching examination was performed to detect the properties about response rate and stability of the polymer during the fast and striking color change applying repeated square-wave potential steps of 4 s between reduced and oxidized states in a 0.2 M TBAPF6 and monomer-free ACN/DCM solution. The polymers were all beforehand synthesized onto ITO-coated glasses with the polymerization charge of 7.41 C/m^2^. The absorption spectrums of the polymers, monitored at the specified wavelengths at which the absorption intensities obviously changed, were revealed in Figure 10, and the parameters of transmittance change, switching time, and coloration efficiency were also obtained.

First of all, no distinct loss emerged during the persistent square wave potential scanning for 300 s, which could indicate the favorable stabilities of the polymer films. Transmittance change (*T*%), also defined as optical contrast, was determined as 7.0% at 375 nm, 11.1% at 615 nm and 50.0% at 1500 nm for P1, and the numerical values of P2, P3 and P4 are all listed in Table 4. As expected, all four polymers had higher *T*% values in the NIR region than that in the visible region, particularly for P4 with a conspicuous 80% contrast at 1660 nm. Table 4 also lists the switching properties of other D-A type polymers, from which it can be seen that the polymers reported in the present manuscript had competitive switching performance, especially in the NIR region, showing fascinating candidates in the application of NIR electrochromic devices.

Switching time, the necessary time to reach 95% of transmittance difference between the neutral state and the oxidized state, was calculated as less than 2 s for P1, P2 and P3, as shown in Table 4, which should be attributed to the rapid dopant ion transport in the doping/de-doping processes. Contrarily, with a shorter switching time than the other three polymers, P4 manifested longer switching time at no matter what test wavelengths, which can be explained by the durability of the P4 film [43].

The coloration efficiency (*CE*), providing the information about the power efficiency of the polymer, is calculated using the optical density (Δ*OD*) and charge consumed per unit electrode area (Δ*Q*) by the following equations:

$$\Delta OD=\log\left(\frac{T_{b}}{T_{c}}\right)\text{ and }CE=\frac{\Delta OD}{\Delta Q}$$

where *T*_b_ and *T*_c_ are the *T*% before and after coloration, respectively. The calculated CE values are also listed in Table 4. All polymers exhibited reasonable coloration efficiency, especially in the NIR region, which confirmed that the four polymers could be applied appropriately as the NIR electrochromic materials. By the above positive analysis, the four polymers displayed rapid charge transport through the coated polymer and the unaltered electroactive during the externally induced potential scanning, which paves the way for utilizing these polymers as electrochromic devices or other materials.

## 4. Conclusions

Four D-A type monomers containing electron rich thiophene derivatives groups as donor units and electron deficient 2,3-bis(4-(decyloxy)phenyl)pyrido[4,3-b]pyrazine groups as acceptor units were synthesized. The corresponding polymers were synthesized by electrochemical polymerization, and the electrochemical and spectroelectrochemical characteristics were investigated. All four polymers displayed n-type doping process and NIR electrochromism with low band gap. The results also showed that the different donor units of the polymers led to remarkable various electrochromic properties. Especially, P3 and P4 with the relatively lower band gap than P1 and P2 exhibited a neutral green color and turned to be transmissive light gray during oxidized process. Furthermore, all the polymers exhibited distinct optical contrast in NIR region, short switching time and excellent coloration efficiency, which revealed their great potential in the applications of high-performance electrochromic device.

## Figures and Tables

**Table 1:** CV data of the polymers.

Polymers	Redox Potential vs. Ag (V)		$E_{p,1/2}^{\mathrm{ox}}$	Redox Potential vs. Ag (V)		$E_{n,1/2}^{\mathrm{ox}}$
	p-Doping	p-De-doping		n-Doping	n-De-doping	
**P1**	1.03	0.95	0.99	−1.17	−1.09	−1.13
	-	-		−0.99	-	
**P2**	1.27	1.11	1.19	−1.15	−1.05	−1.10
	-	-		−0.98	-	
**P3**	0.92	0.76	0.84	−1.22	−1.06	−1.14
	0.22	-		−1.00	-	
**P4**	0.88	0.77	0.825	−1.27	−1.15	−1.21
	0.46	-		−1.12	-	

**Table 2:** The onset oxidation potential (*E*_onset_), absorption onsets wavelength (λ_onset_), optical band gap (*E*_g_), HOMO and LUMO energy levels and electrochemical band gaps of the monomers and the corresponding polymers.

Compound	*E*_onset_ vs. Ag (V)	λ_onset_ (nm)	*E*_g,op_^(a)^ (eV)	HOMO ^(b)^ (eV)	LUMO ^(c)^ (eV)	*E*_g,cal_^(d)^ (eV)	*E*_g,ec_^(e)^ (eV)
**M1**	1.15	523	2.37	−5.57	−3.20	2.84	-
**M2**	1.15	543	2.28	−5.57	−3.29	2.79	-
**M3**	0.95	550	2.25	−5.37	−3.12	2.73	-
**M4**	0.77	576	2.15	−5.19	−3.04	2.70	-
**P1**	0.69	844	1.47	−5.11	−3.64	-	1.48
**P2**	0.93	870	1.42	−5.35	−3.93	-	1.87
**P3**	0.02	1002	1.24	−4.44	−3.20	-	1.27
**P4**	0.15	1342	0.92	−4.57	−3.65	-	1.45
**P(A) ^(f)^**	-	-	1.14	-	-	-	-
**P(B) ^(g)^**	-	-	1.4	-	-	-	-

^(a)^ *E*_g,op_ = 1241/λ_onset_; ^(b)^ HOMO = −e(*E*_onset_ + 0.02 + 4.4) (vs. SCE), in which the number 0.02 is to calibrate the error of reference electrode; ^(c)^ LUMO = −|HOMO − *E*_g**,**op_|, where HOMO and *E*_g_ refer to the absolute value; ^(d)^ *E*_g,cal_ was calculated by DFT calculations; ^(e)^ *E*_g,ec_ was calculated from the onset potentials for p- and n-type doping of the polymers; ^(f)^ See the reference [17]; ^(g)^ See the reference [18].

**Table 3:** The colorimetry analyses of the polymers.

Polymers	*E*, vs. Ag (V)	L*	a*	b*	Color
**P1**	0	46	−2	−7	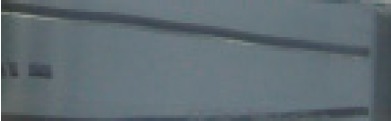
	1.4	49	−1	2	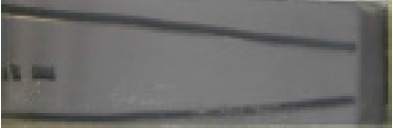
**P2**	0	37	−8	−40	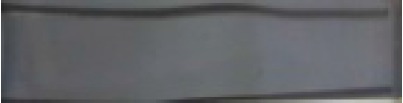
	1.4	60	−12	8	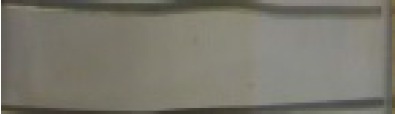
**P3**	0	44	−62	−20	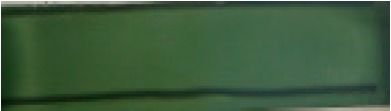
	1.2	56	−2	3	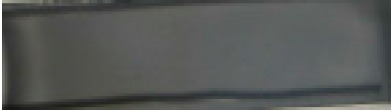
**P4**	−0.4	60	−40	16	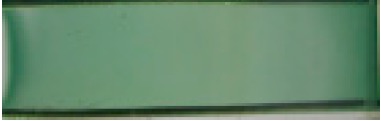
	1.1	62	−12	8	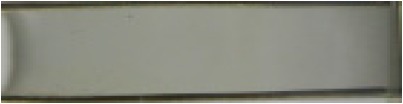

**Table 4:** The switching properties of the polymers.

Polymer	λ (nm)	Optical contrast (%)	Response time (s)	Coloration efficiency (cm^2^·C^−1^)
**P1**	375	7.0	0.92	77.00
	615	11.1	0.70	72.97
	1,500	50.0	1.35	297.61
**P2**	415	10.0	0.62	78.45
	556	14.5	0.70	82.67
	1,550	61.5	1.80	323.25
**P3**	408	11.1	1.30	80.65
	830	17.0	0.81	98.88
	1,630	60.2	1.40	279.50
**P4**	422	18.6	3.33	106.76
	754	17.1	2.42	106.72
	1,660	80.0	2.20	340.15
**P(A) ^(a)^**	408	16.7	0.61	87
	820	25.9	0.55	161.8
	2,000	61.3	0.35	309.6
**P(B) ^(b)^**	1,490	50.0	-	-
	2,000	18.0		

^(a)^ See the reference [17]; ^(b)^ See the reference [18].

## Supplementary Materials

The following are available online at www.mdpi.com/2073-4360/8/10/377/s1. Figure S1: (a) 1H NMR spectrum of 2,5-diromopyrido-3,4-diamine (1) in DMSO. Solvent peak at δ = 2.49 ppm is marked by “x”, water peak at δ = 3.33 ppm is marked by “y”; (b) ^13^C NMR spectrum of 1 in DMSO. Solvent peak at δ = 40.76 ppm is marked by “X”. Figure S2: (a) ^1^H NMR spectrum of 1,2-bis(4-(decyloxy)phenyl)ethane-1,2-dione (2) in CDCl_3_. Solvent peak at δ = 7.26 ppm is marked by “x”; (b) ^13^C NMR spectrum of 2 in CDCl_3_. Solvent peak at δ = 72.50 ppm is marked by “X”. Figure S3: (a) ^1^H NMR spectrum of 5,8-dibromo-2,3-bis(4-(decyloxy)phenyl)pyrido[4,3-b]pyrazine (3) in CDCl_3_. Solvent peak at δ = 7.26 ppm is marked by “x”; (b) ^13^C NMR spectrum of 3 in CDCl_3_. Solvent peak at δ = 72.50 ppm is marked by ”X”. Figure S4: (a) ^1^H NMR spectrum of M1 in CDCl_3_. Solvent peak at δ = 7.26 ppm is marked by “x”; (b) ^13^C NMR spectrum of M1 in CDCl_3_. Solvent peak at δ = 72.50 ppm is marked by “X”. Figure S5: (a) ^1^H NMR spectrum of M2 in CDCl_3_. Solvent peak at δ = 7.26 ppm is marked by “x”; (b) ^13^C NMR spectrum of M2 in CDCl_3_. Solvent peak at δ = 72.50 ppm is marked by “X”. Figure S6: (a) ^1^H NMR spectrum of M3 in CDCl_3_. Solvent peak at δ = 7.26 ppm is marked by “x”; (b) ^13^C NMR spectrum of M3 in CDCl_3_. Solvent peak at δ = 72.50 ppm is marked by “X”. Figure S7: (a) ^1^H NMR spectrum of M4 in CDCl_3_. Solvent peak at δ = 7.26 ppm is marked by “x“; (b) ^13^C NMR spectrum of M4 in CDCl_3_. Solvent peak at δ = 72.50 ppm is marked by “X“. Figure S8: Cyclic voltammetry (CV) curves of the monomers: (a) M2; (b) M3; and (c) M4. Figure S9: CV curves of the polymers for p-type doping process at various scan rates: (a) P2; (b) P3; and (c) P4. Insert: graphs of scan rate vs. peak current density. Figure S10: The first and the 1000th CV curve of the polymers: (a) P2; (b) P3; and (c) P4. Figure S11: Film thicknesses of the polymers deposited potentiostatically onto ITO electrode: (a) P1; (b) P2; (c) P3; and (d) P4.
