# Supplementary material for: Low Band Gap Donor–Acceptor Type Polymers Containing 2,3-Bis(4-(decyloxy)phenyl)pyrido[4,3-b]pyrazine as Acceptor and Different Thiophene Derivatives as Donors
Source: Polymers (Basel). 2016 Oct 24;8(10):377. doi: 10.3390/polym8100377 (PMC6432059; doi:10.3390/polym8100377)
Supplement: Supplementary file 1 [file polymers-08-00377-s001.pdf]

Supplementary Materials: Low Band Gap Donor-Acceptor Type Polymers Containing 2,3-Bis(4-(decyloxy)phenyl)pyrido[4,3-b]pyrazine as Acceptor and Different Thiophene Derivatives as Donors

Yan Zhang, Xuezhong Liu, Min Wang, Xiaoli Liu and Jinsheng Zhao

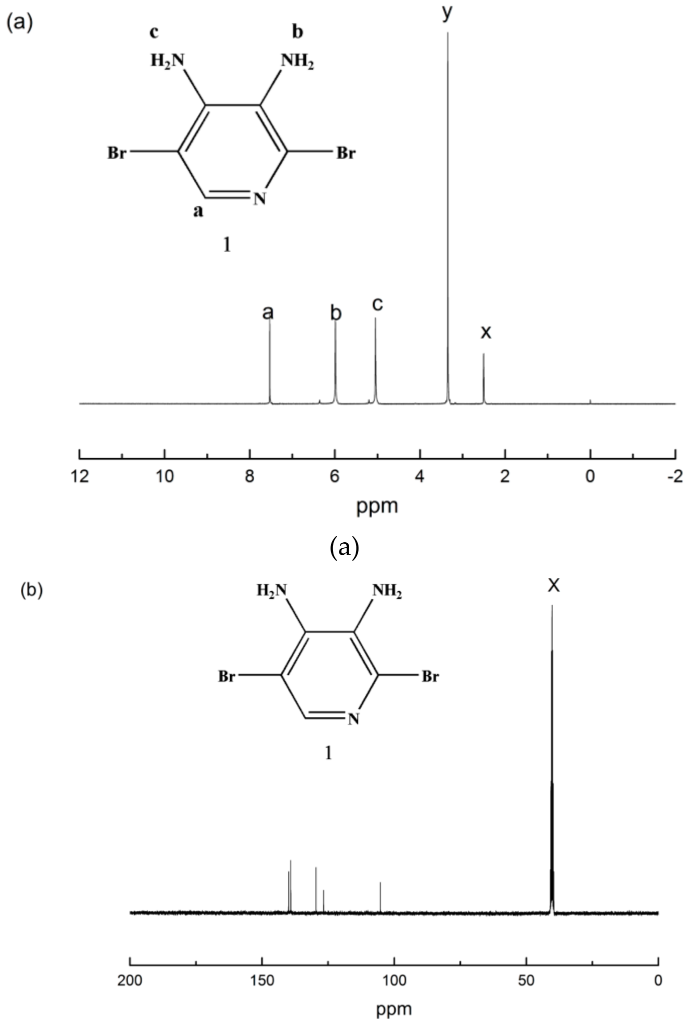

Figure S1. (**a**) ^{1}H NMR spectrum of 2,5-diromopyrido-3,4-diamine (**1**) in DMSO. Solvent peak at δ = 2.49 ppm is marked by "x", water peak at δ = 3.33 ppm is marked by "y"; (**b**) ^{13}C NMR spectrum of **1** in DMSO. Solvent peak at δ = 40.76 ppm is marked by "X".

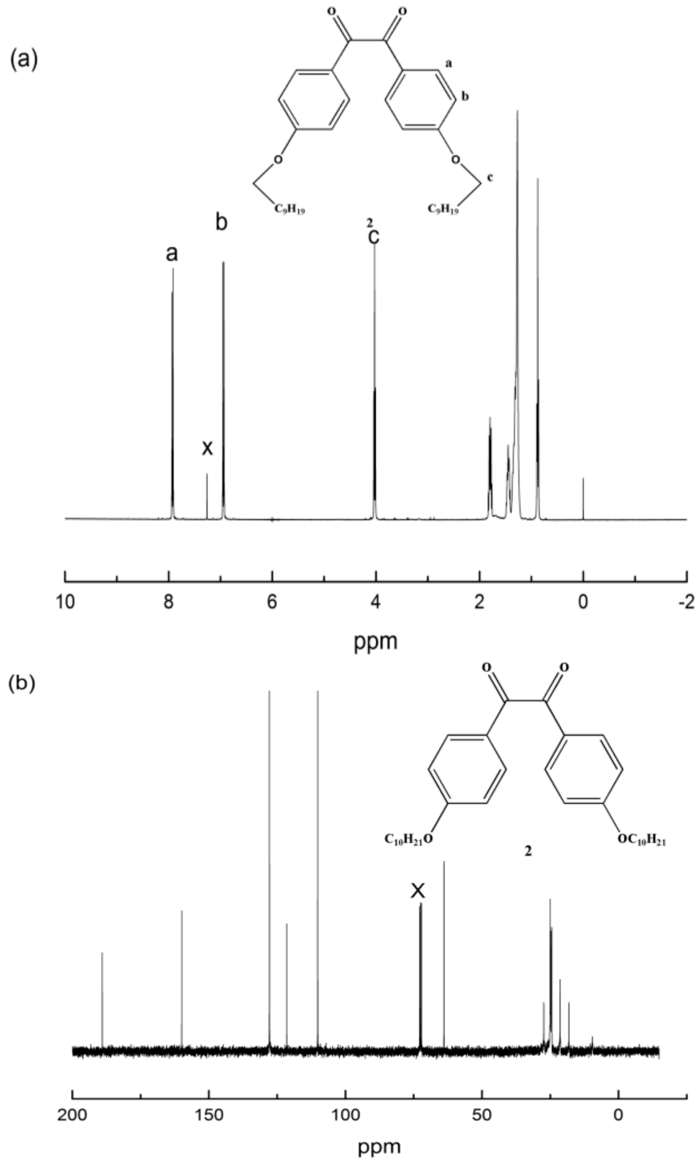

Figure S2. (**a**) ^{1}H NMR spectrum of 1,2-bis(4-(decyloxy)phenyl)ethane-1,2-dione (**2**) in $CDCl_3$. Solvent peak at $\delta = 7.26$ ppm is marked by "x"; (**b**) ^{13}C NMR spectrum of **2** in $CDCl_3$. Solvent peak at $\delta = 72.50$ ppm is marked by "X".

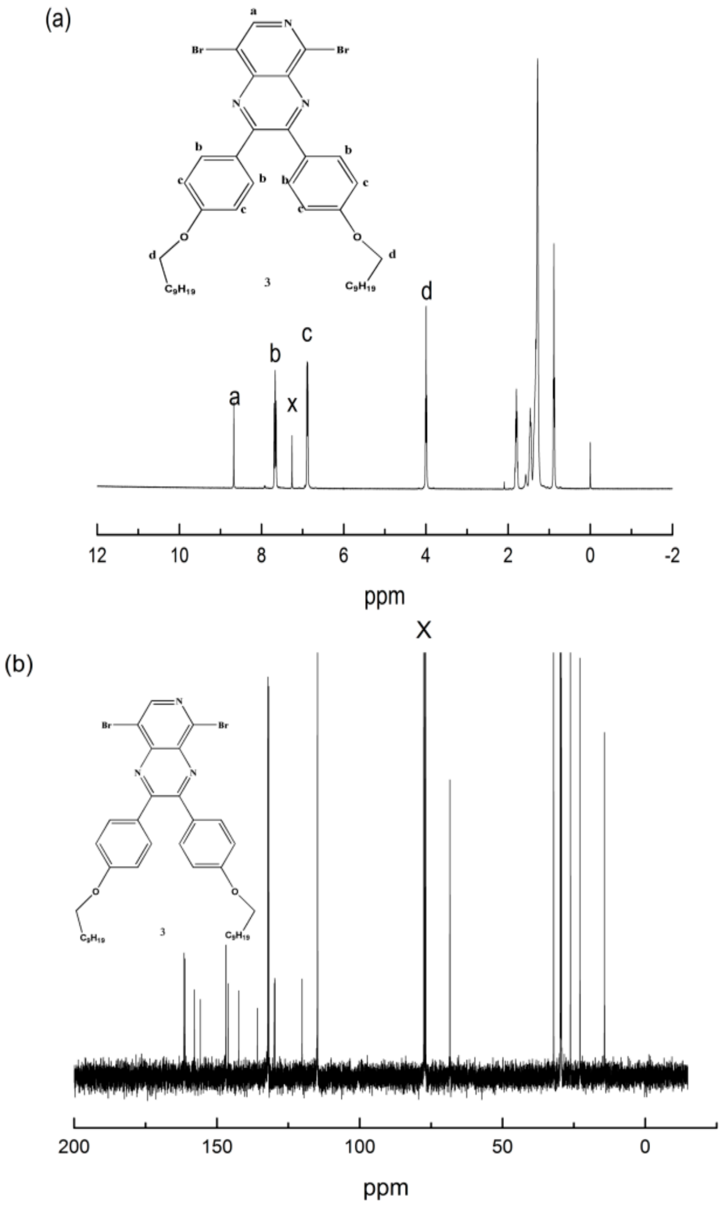

Figure S3. (**a**) ^{1}H NMR spectrum of 5,8-dibromo-2,3-bis(4-(decyloxy)phenyl)pyrido[4,3-b]pyrazine (**3**) in $CDCl_3$. Solvent peak at δ = 7.26 ppm is marked by "x"; (**b**) ^{13}C NMR spectrum of **3** in $CDCl_3$.Solvent peak at δ = 72.50 ppm is marked by "X".

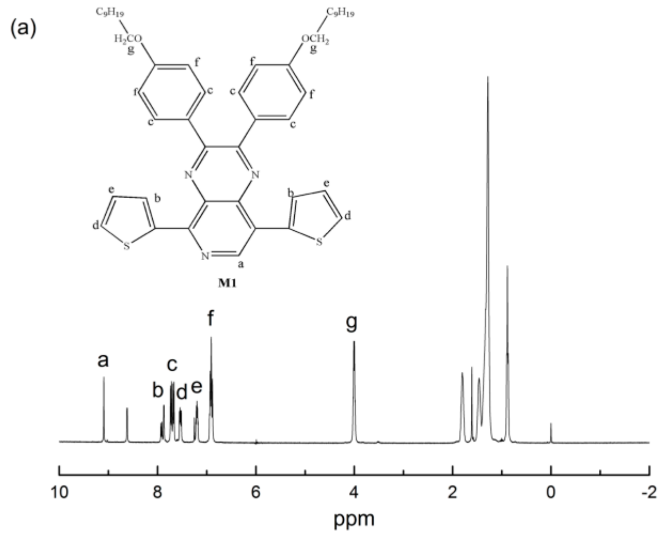

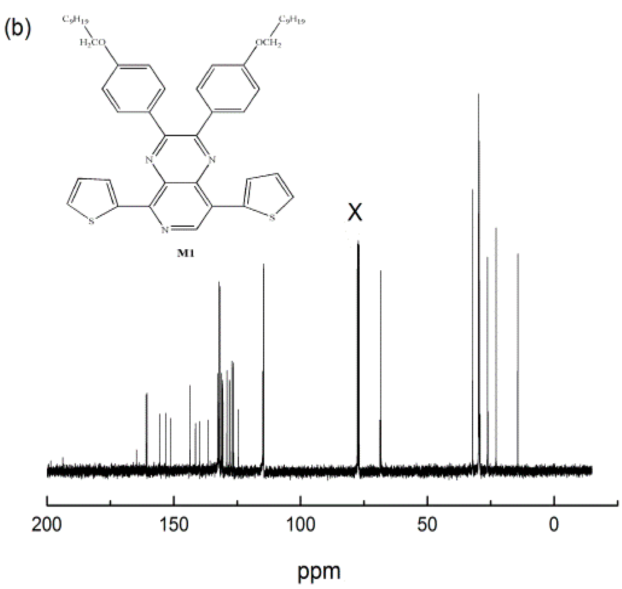

Figure S4. (**a**) ^{1}H NMR spectrum of M1 in $CDCl_3$. Solvent peak at δ = 7.26 ppm is marked by "x"; (**b**) ^{13}C NMR spectrum of M1 in $CDCl_3$. Solvent peak at δ = 72.50 ppm is marked by "X".

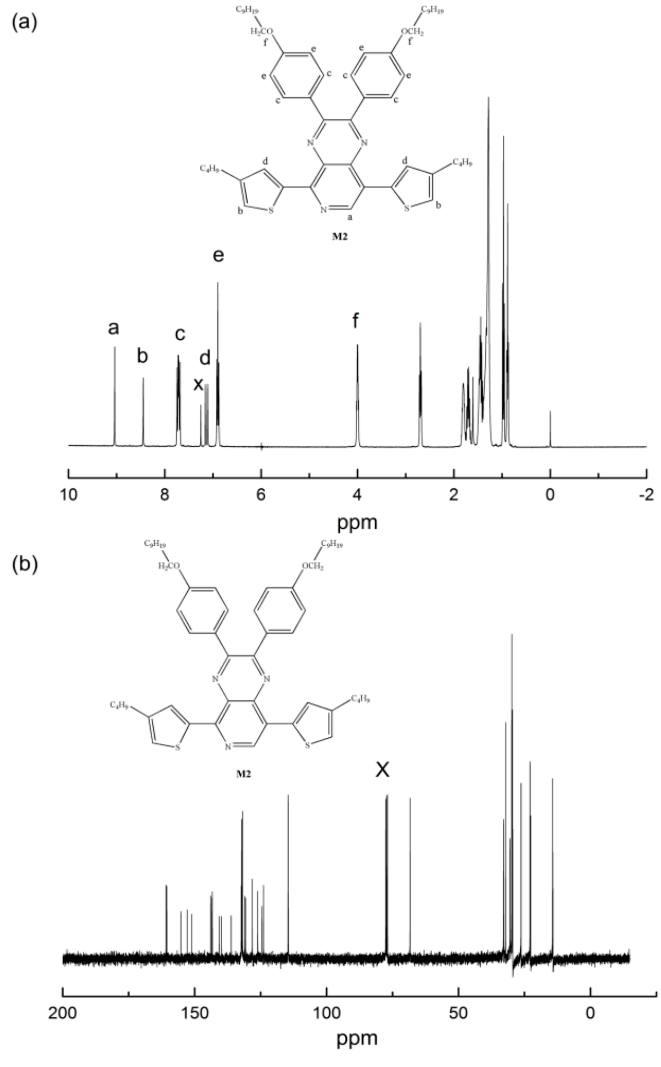

Figure S5. (**a**) ^{1}H NMR spectrum of M2 in $CDCl_3$. Solvent peak at δ = 7.26 ppm is marked by "x"; (**b**) ^{13}C NMR spectrum of M2 in $CDCl_3$. Solvent peak at δ = 72.50 ppm is marked by "X".

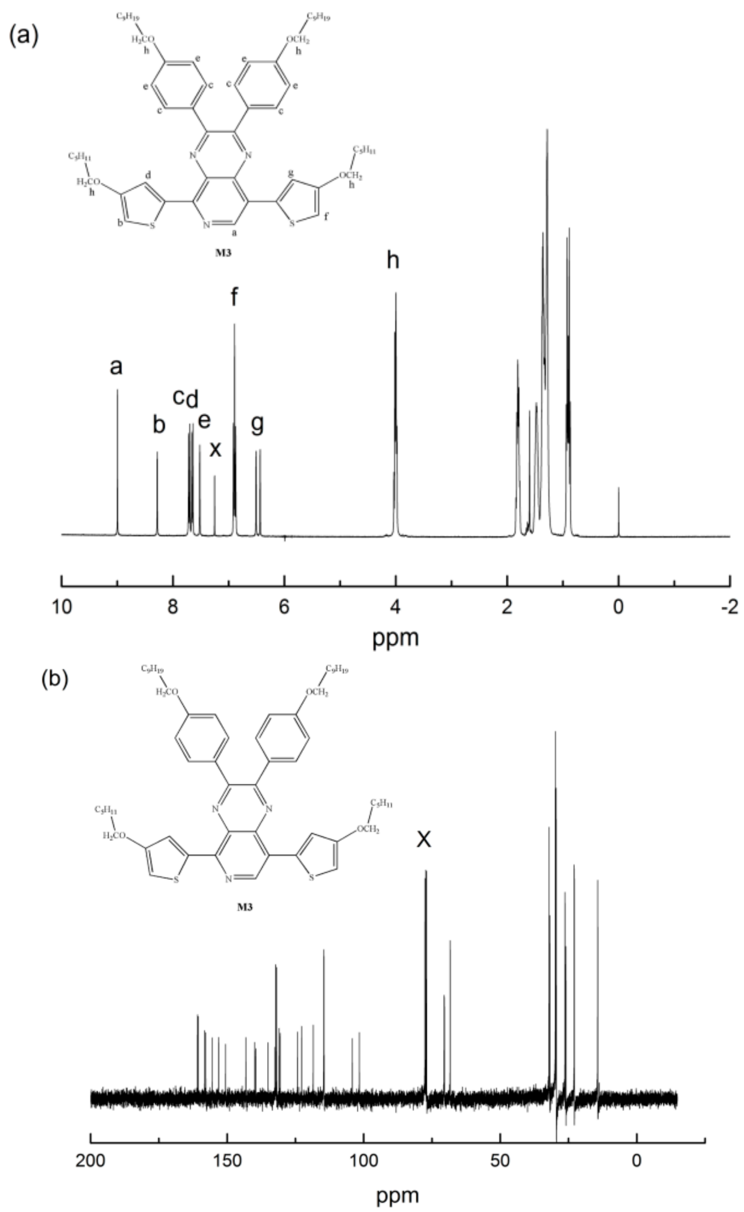

Figure S6. (**a**) ^{1}H NMR spectrum of M3 in $CDCl_3$. Solvent peak at δ = 7.26 ppm is marked by "x"; (**b**) ^{13}C NMR spectrum of M3 in $CDCl_3$. Solvent peak at δ = 72.50 ppm is marked by "X".

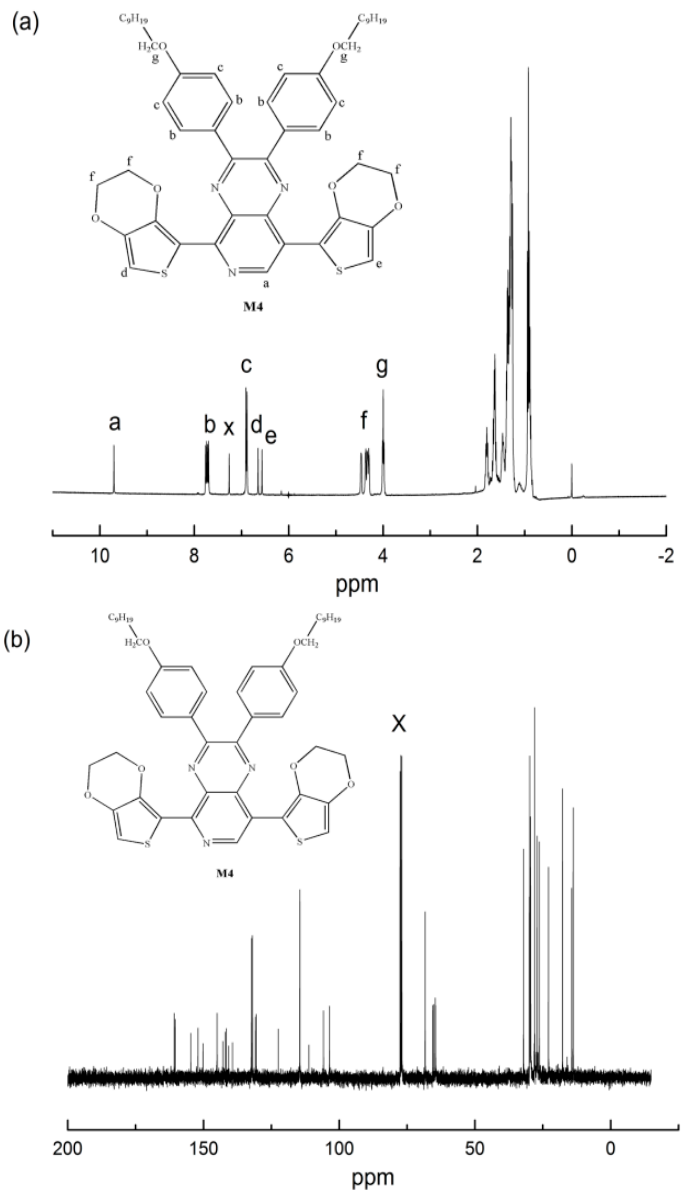

Figure S7. (**a**) ^{1}H NMR spectrum of M4 in $CDCl_3$. Solvent peak at δ = 7.26 ppm is marked by "x"; (**b**) ^{13}C NMR spectrum of M4 in $CDCl_3$. Solvent peak at δ = 72.50 ppm is marked by "X".

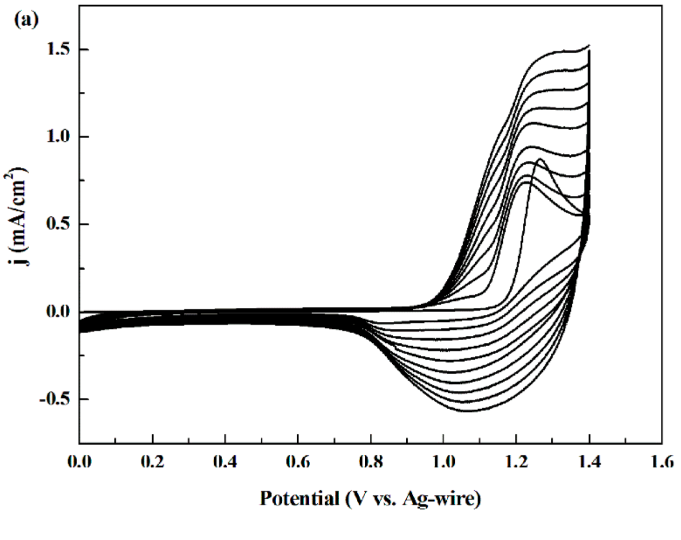

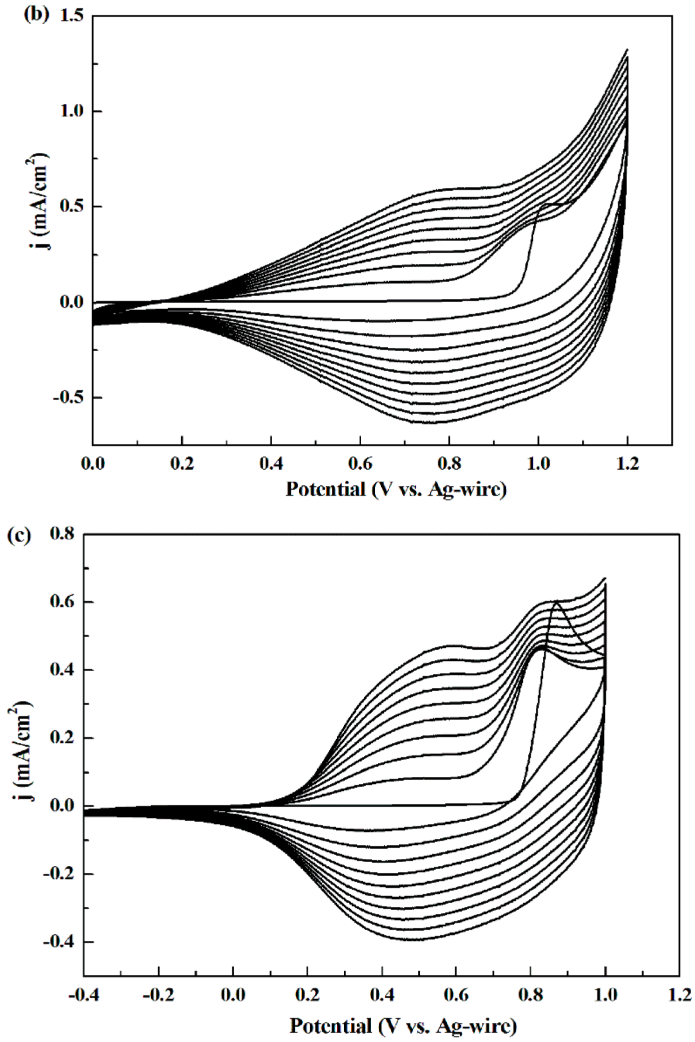

Figure S8. Cyclic voltammetry (CV) curves of the monomers: (**a**) M2; (**b**) M3; and (**c**) M4.

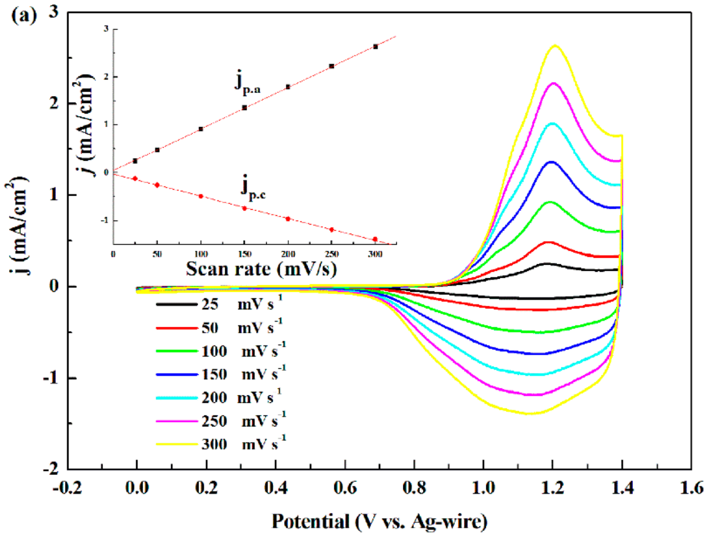

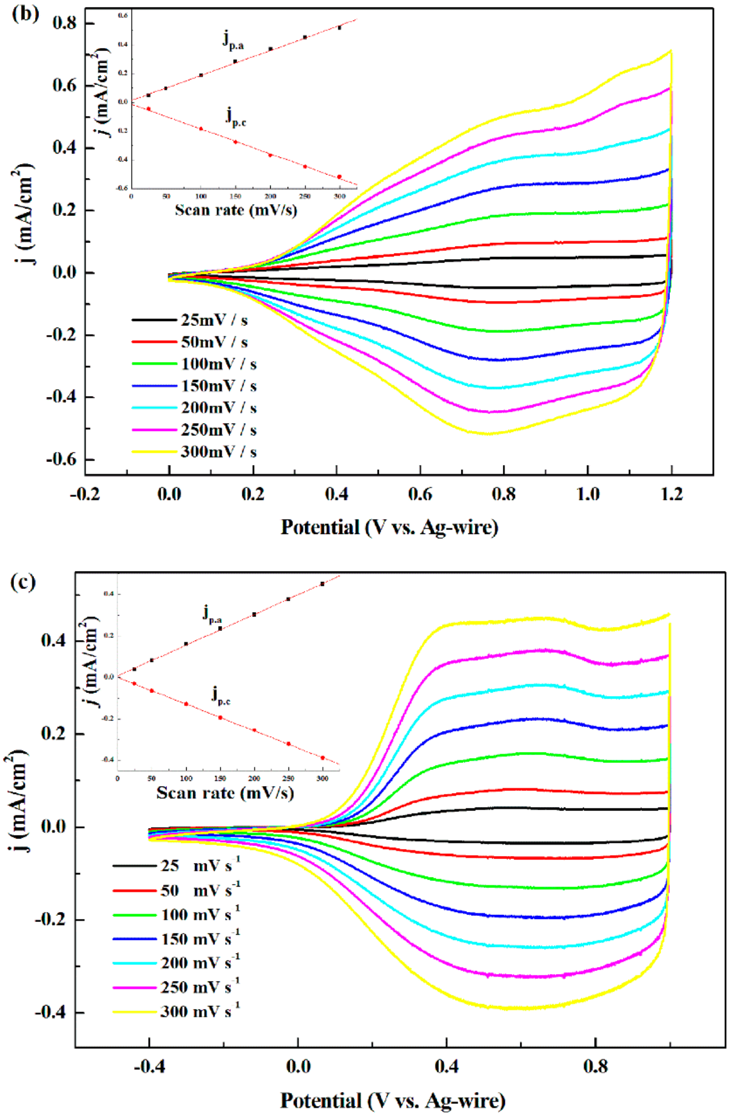

Figure S9. CV curves of the polymers for p-type doping process at various scan rates: (**a**) P2; (**b**) P3; and (**c**) P4. Insert: graphs of scan rate vs. peak current density.

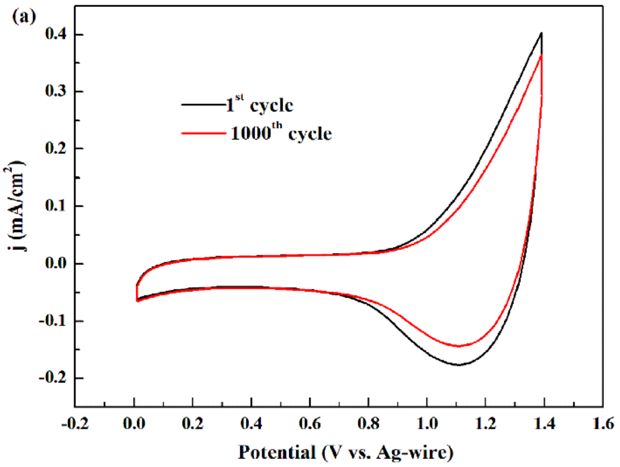

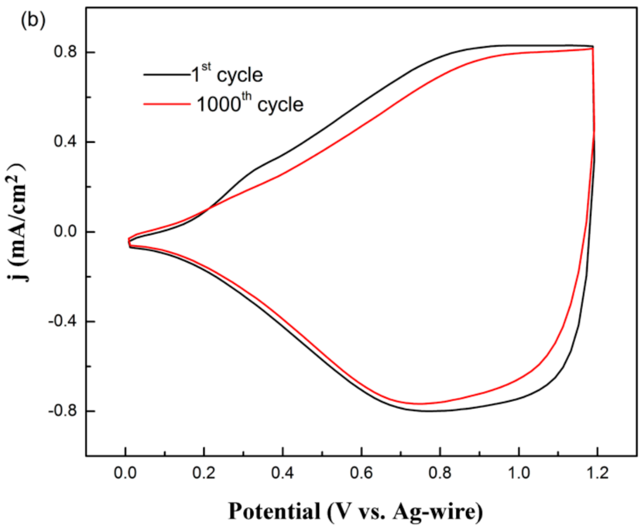

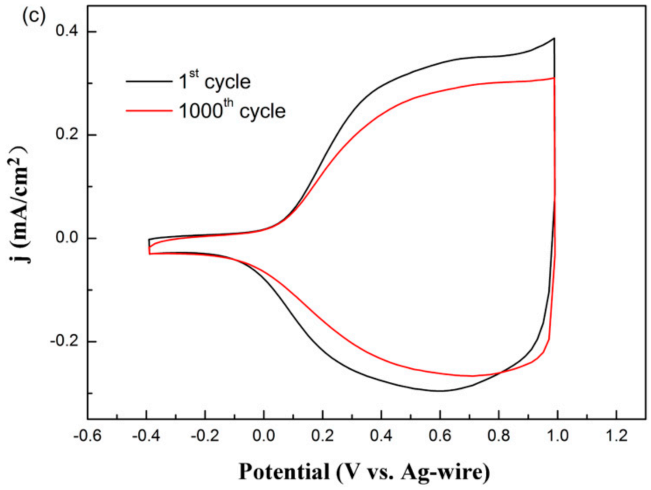

Figure S10. The first and the 1000th CV curve of the polymers: (**a**) P2; (**b**) P3; and (**c**) P4.

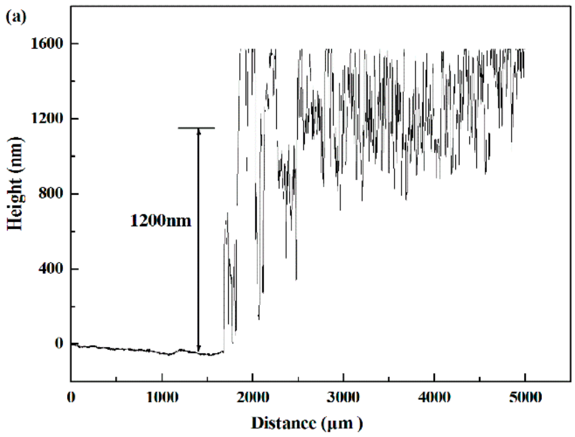

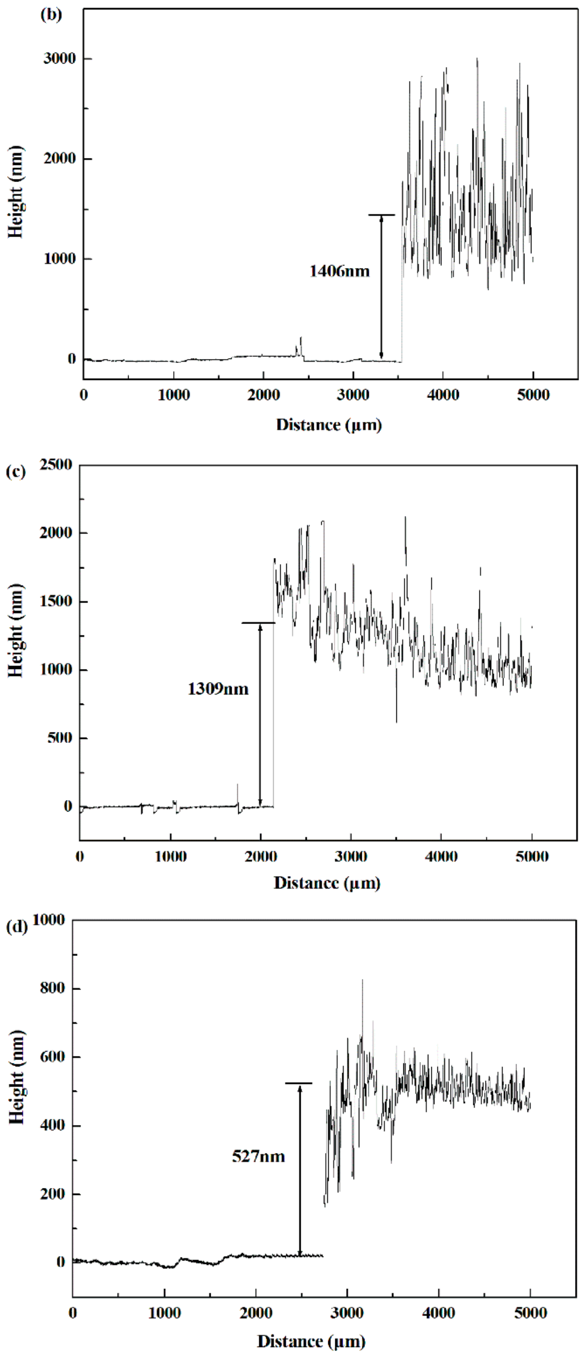

Figure S11. Film thicknesses of the polymers deposited potentiostatically onto ITO electrode: (**a**) P1; (**b**) P2; (**c**) P3; and (**d**) P4.
